# The Effect of Synthesis Conditions and Chemical Structure of Thermoplastic Polyimides on Their Thermomechanical Properties and Short-Term Electrical Strength

**DOI:** 10.3390/polym17101385

**Published:** 2025-05-18

**Authors:** Victor M. Nazarychev, Andrey A. Pavlov, Almaz M. Kamalov, Margarita E. Borisova, Andrei L. Didenko, Elena M. Ivan’kova, Vadim E. Kraft, Gleb V. Vaganov, Alexandra L. Nikolaeva, Anna S. Ivanova, Victor K. Lavrentiev, Elena N. Popova, Ivan V. Abalov, Aleksey N. Blokhin, Alexander N. Bugrov, Vladislav V. Kudryavtsev

**Affiliations:** 1 Branch of Petersburg Nuclear Physics Institute Named by B.P. Konstantinov of National Research Centre «Kurchatov Institute»—Institute of Macromolecular Compounds, Bolshoi, Pr. 31 (V.O.), 199004 St. Petersburg, Russia; spb.kamalov@gmail.com (A.M.K.); ivelen@mail.ru (E.M.I.); sparta3006@inbox.ru (V.E.K.); glebvaganov@mail.ru (G.V.V.); alexandra.l.nikolaeva@gmail.com (A.L.N.); annest.2107@yandex.ru (A.S.I.); lavrentev1949@mail.ru (V.K.L.); popovaen@hq.macro.ru (E.N.P.); i.abalf@yandex.ru (I.V.A.); blokhin_an@hq.macro.ru (A.N.B.); bugrov.an@mail.ru (A.N.B.); kudryav@hq.macro.ru (V.V.K.); 2 Institute of Energy, Peter the Great St Petersburg Polytechnic University, The Politekhnicheskaya St. 29, 195251 St. Petersburg, Russia; pavlov.aa.hv@mail.ru (A.A.P.); vladimirl.borisov@gmail.com (M.E.B.); 3 Department of Physical Chemistry, Saint Petersburg Electrotechnical University (ETU “LETI”), ul. Professora Popova 5, 197376 St. Petersburg, Russia

**Keywords:** polyimides, thermophysical properties, mechanical properties, short-term electrical strength

## Abstract

Polyimides (PIs) are materials that are resistant to high temperatures and crucial for the manufacturing of films, fibers, coatings, and 3D-printed items. PIs are widely used as electrically insulating materials in electronics and electrical engineering. This study investigated how the chemical structure (i.e., choice of initial monomers), the synthesis conditions of the prepolymer (i.e., choice of amide solvent), and the conditions for forming polyimide films (i.e., final curing temperature) affect the thermophysical properties and short-term electrical strength of obtained polyimide films of different chemical structures. In this work, we varied the compositions of the dianhydrides used for synthesizing polyamic acids—pyromellitic acid (PMDA), tetracarboxylic acid diphenyl oxide (ODPA) and 1,3-bis(3′,4-dicarboxyphenoxy)benzene acid (R)—with a constant diamine: 4,4′-oxydianiline (ODA). Additionally, we varied the amide solvents employed: *N*,*N*-dimethylacetamide (DMAc), *N*,*N*-dimethylformamide (DMF), and *N*-methyl-2-pyrrolidone (NMP). This study represents the first investigation into how the choice of solvent in the synthesis of thermoplastic polyimide prepolymers affects their short-term electrical strength. The molecular weights of the polyamic acids were determined using gel permeation chromatography (GPC). The deformation and strength characteristics of the investigated films were also assessed. The thermophysical properties of the polyimides were evaluated via dynamic mechanical analysis (DMA), differential scanning calorimetry (DSC), and thermogravimetric analysis (TGA). X-ray diffraction analysis and infrared spectroscopy (IR) were conducted on the examined film samples. The short-term electrical strength was also evaluated.

## 1. Introduction

Aromatic polyimides (PIs) are widely used high-tech materials known for their high thermo-oxidative stability and strength [1,2,3,4,5,6,7]. Materials based on aromatic polyimides are of interest as products that operate over a wide temperature range (from −250 °C to +350 °C and above), exhibit resistance to high levels of radiation, and withstand aggressive chemical solvents [1,2,3,4,5,6,7]. PIs serve as high-temperature binders in the fabrication of structural materials [8,9,10,11]. PIs are used on an industrial scale for manufacturing electrically insulating coatings and films in electrical and electronics engineering [12,13,14,15,16,17,18,19,20]. The most common polyimide utilized for electrical insulation is non-thermoplastic poly(4,4′-oxydiphenylene pyromellitimide) (PMDA-ODA), known as the industrial film brand Kapton^®^ [21,22]. However, PMDA-ODA polyimide exhibits poor adhesion to metals, high hygroscopicity, and limited recyclability. In this context, PI thermoplastics are of interest as potential materials for modern 3D printing technologies aimed at the fabrication of electrically insulating materials [23]. The main design direction of thermoplastic polyimides is to enrich the repeating unit of the polymer with bridging (hinge) atoms or groups, as well as lateral bulky substituents [6], which enhances chain mobility and affects the glass transition temperature *T_g_* [24,25,26]. The first work on the introduction of polyimide thermoplastics as electrical insulation materials dates back to the late 1990s [14]. A more recent study [15] shows that the PI film brand Ultem^TM^ [27] is promising as an electrical insulation coating for interturn insulation in power transformers. There is a growing interest in new polyimide coatings that demonstrate increased electrical strength [15,16,17,18,19] compared to materials on the market. In addition, polymer composite materials where the filler affects the electrical strength of the insulating material present a technological perspective [20]. In this study, the introduction of ZnO filler into a polymer matrix increased electrical strength by almost 50% compared to an unfilled polymer. Zhang et al. [28] incorporated aluminum nanoclusters into PIs, which resulted in an increase in their electrical strength 1.5-fold. Dong et al. [29] conducted a study on polyimide composite films containing various types and quantities of graphene, resulting in a significant increase in electrical strength of up to nearly 70%. In their work, the composite electrical strength was regulated by the amount of modified graphene introduced into the polyimide. There is considerable interest in the preparation of electrically strong materials based on thermoplastic PIs using modern plastic processing technologies.

Owing to the importance of PIs as electrical insulating materials, there is a considerable amount of literature on their electrical strength without losing their dielectric properties [14,15,16,17,18,19,20]. The dielectric properties of PIs, apart from breakdown strength, are determined by their chemical structure, synthesis conditions, and curing temperatures. Wang et al. [30] concluded that the inclusion of polar functional groups increases the dielectric constant via dipolar polarization, whereas polarizable groups at the electrode–monomer interface boost the permittivity and loss. Tanikella et al. [31] found that solvents can increase the dielectric loss, especially when the curing process occurs quickly. They demonstrated that lower curing temperatures can preserve solvents, which in turn increases dielectric loss. Conversely, Chu et al. [32] showed that higher temperatures promote cross-linking and decrease dielectric loss by removing solvents. However, a study on the influence of all considered factors on the dielectric properties of thermoplastic polyimides was out of scope of the current research and will be a topic of future work. Unfortunately, studies on the electrical strength of thermoplastic PIs have not been comprehensively covered in the literature. Investigating the electrical strength of PI films, particularly those made from PMDA-ODA, is essential because of their potential use in capacitors operating at high temperatures. PMDA-ODA films exhibit enhanced dielectric properties; however, their practical application in capacitors is hindered by moisture absorption, which can lead to dielectric breakdown. In traditional PIs, nanopores that trap moisture can form and reduce the electrical strength of the dielectric material [33,34]. Although polarization processes have been studied to improve dielectric performance and short-term electrical strength, these materials remain unsuitable for capacitor fabrication [35,36]. The use of thermoplastic PIs opens up promising technologies for their production and processing, including injection molding and 3D printing. These technologies will improve control over the microstructure of the material, facilitating the removal of nanopores, which reduce the electrical strength of films obtained with solution casting [37,38]. Thermoplastic PIs can possess tailored thermal and mechanical properties exceeding those of traditional polyimide materials owing to their improved PI processing capabilities and stable dielectric characteristics [39,40]. Obtaining PIs without nanopores can enhance the reliability and lifetime of capacitors in electronic devices with higher requirements [41]. It is essential to replace thermosetting PIs with thermoplastic PIs, as their electrical properties, which include specific conductivity and short-term electrical strength, must be comparable to or better than those of Kapton HN [42]. It was found [43] that the unique electrical conductivities of PIs remain unaffected by *T_g_* at temperatures lower than *T_g_*. The objective of this study was to elevate electrical strength to a value of at least 240 kV/mm.

There is also a gap in the literature regarding computer simulations of the electrophysical properties of thermoplastic PIs, which is necessary for selecting polyimides with optimal performance properties. The ultimate objective of this research was to ascertain the short-term electrical toughness of thermoplastic PIs, thereby enabling the selection of suitable PI matrices for fabricating both standalone coatings and composite electrical insulating materials.

## 2. Materials and Methods

### 2.1. Materials

Reagents serving as initial monomers for the synthesis of the examined PIs were: pyromellitic acid dianhydride (PMDA) (Aldrich, UK, 99%, CAS#: 89-32-7); 3,3′,4,4′-oxydiphthalic anhydride (ODPA) (Aldrich, UK, 98%, CAS#: 2421-28-5); 4,4′-oxydianiline (ODA) (Tokyo Chemical Industry, Japan, 98%, CAS#: 101-80-4); and 1,3-bis(3′,4-dicarboxyphenoxy)benzene (dianhydride R) (TechKhimProm LLC, Yaroslavl, Russia, with a melting temperature of approximately 164 °C). The solvents *N*,*N*-dimethylacetamide (DMAc) (J-SC Vecton, Yekaterinburg, Russia, Chemical Grade), *N*,*N*-dimethylformamide (DMF) (J-SC Vecton Russia, Chemical Grade), and *N*-methyl-2-pyrrolidone (NMP) (J-SC Vecton Russia, Chemical Grade) were dehydrated using calcium hydride and subjected to two distillations under an argon atmosphere.

### 2.2. Experimental Techniques

The chemical structure of PIs was confirmed using IR spectroscopy on a Vertex 70 FT-IR spectrometer (Bruker, Germany) equipped with a MIRacle-attenuated full internal reflectance (ATR) micro-attachment (Pike Technologies, Fitchburg, WI, USA).

To determine the molecular weight characteristics, the polyamic acid (polyimide prepolymer) solutions were analyzed using a Shimadzu LC-20 Prominence instrument, which was equipped with a Waters Styragel HT4 column measuring 7.8 × 300 mm and a particle diameter of 10 μm, as well as a Shimadzu RID-20A refractometer detector. The eluent used was a solution of *N*,*N*-dimethylformamide, which was 99.9% pure for high-performance liquid chromatography (HPLC) and supplied by Aldrich, and contained 0.1 mol/L anhydrous lithium chloride (LiBr) and 0.06 mol/L phosphoric acid. The eluent was used at a temperature of 60 °C and a flow rate of 1.0 mL/min. The molecular masses were calculated by a calibration relationship established with the PSS ReadyCal Kit, which utilized PEO (polyethylene oxide) standards with molecular weights ranging from 6 to 1500 kDa.

The thermophysical properties of the samples were studied using complex thermal analysis. Thermogravimetric analysis of the samples was carried out on a TG 209 F1 thermal analyzer (Neitch, Germany). The tests were carried out in a temperature range of 30 to 800 °C with a heating rate of 20 °C/min in an inert medium (argon). Each sample had a mass of 2–3 mg. The results of the TGA experiment were as follows: residual mass at 800 °C and temperatures corresponding to 5% and 10% mass loss (τ_5_, τ_10_).

The glass and phase transition temperatures for the synthesized PIs were determined using a DSC 204 F1 Phoenix calorimeter (Netzsch, Germany). Differential scanning calorimetry (DSC) was carried out in a temperature range of 25 to 400 °C at a heating rate of 10 °C/min in an inert medium (argon). Each sample weighed 3–4 mg. From the DSC experiments, the *T_g_* values of the samples were determined from the second thermal change scan.

The temperature dependence of the elastic modulus *E*′ and the tangent loss angle tgδ of the polymeric materials was measured by dynamic mechanical analysis (DMA) on a DMA 242 C instrument (Netzsch, Germany). The *T_g_* value was determined from the high-temperature maximum of the tgδ curves. The measurements were carried out at a frequency of 1 Hz while increasing the temperature from 25 to 450 °C, with a temperature increase rate of 5 °C/min and a fixed strain amplitude of 0.1%.

The stress–strain characteristics of the polymer samples were determined using universal tensile machines Instron 5943 (UK) and AGS-X 5kN (Shimadzu, Kyoto, Japan) in uniaxial tensile mode at room temperature. The measurements were carried out following ASTM D638 requirements. The tensile speed was set to 10 mm/min. The samples measured 40 mm in length and 2 mm in width, with a working length of 20 mm. For each sample, 6 to 8 film strips were tested, and the average values for all specified parameters were calculated. Young’s modulus, strength, and strain at break were derived from the obtained tensile diagrams.

Wide-angle X-ray scattering (WAXS) studies of model film samples were conducted on an automated XR DRON-2.0 diffractometer manufactured by Leningrad NPO Burevestnik. Cu-Kα radiation was employed in this study. Monochromatization of X-ray radiation was carried out using a Ni filter. The WAXS diffractograms were recorded in transmission.

The densities of the model films were determined using the flotation method at room temperature with small fragments of samples in mixtures of carbon tetrachloride and toluene [44].

The breakdown of the PI samples was performed using a SKAT-70 high-voltage testing apparatus. The electrical strength was determined at an AC voltage frequency of 50 Hz, increasing at a rate of 0.5 kV/s until dielectric breakdown. The tests were performed at room temperature. The dimensions of the laboratory production were 9 × 9 cm. Taking this into account, the breakdown tests were carried out in a plane-ball electrode system. The diameter of the ball was 4 mm. To eliminate edge partial discharges, the samples were immersed in transformer oil. The dielectric breakdown was recorded as a voltage drop across the sample. The error in voltage measurement was 2.6%.

### 2.3. Objects of Study

**Figure 1:**
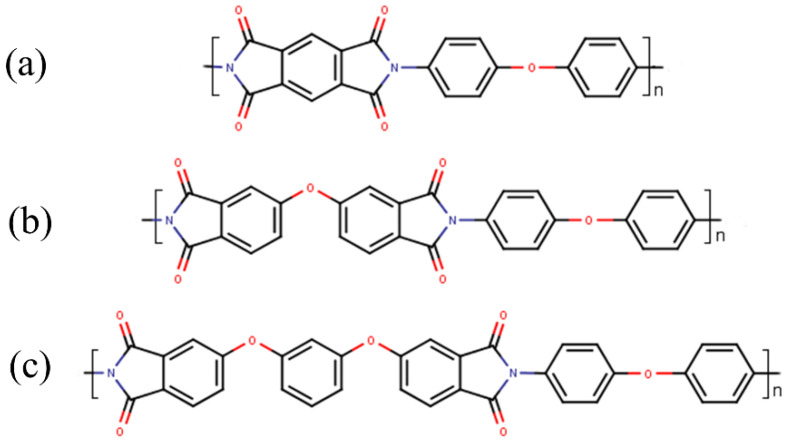
Chemical structures of the polyimide repeating units: (**a**) PMDA-ODA, (**b**) ODPA-ODA, and (**c**) R-ODA.

The chemical formulas of the PIs investigated in this study are presented in Figure 1.

### 2.4. Chemical Synthesis of Polyamic Acids

The synthesis of prepolymers in the form of polyamic acids (PAA) was carried out according to methods described in the literature [2,3,4,5,6,7].

A total of 5.0 g (25 mmol) of 4,4′-oxydianiline was placed in a three-neck flask equipped with a mechanical stirrer and a Drexler nozzle for the argon inlet and outlet. After dissolving the diamine with an aprotonic solvent (DMAc, DMF, or NMP) at 15 °C, 25 mmol of one of the three dianhydrides (PMDA, ODPA, or R) was added. After six hours of vigorous stirring at 15 °C, nine polyamic acid (PAA) samples were synthesized, specifically R-ODA, PMDA-ODA, and ODPA-ODA, each in the three amide solvents employed. These samples exhibited weight concentrations ranging from 15% to 20%, and were selected to optimize the molecular weight of the PAAs. Subsequently, the samples were filtered through a Nutch filter at a pressure of 10 atm using a metal filtration membrane with a pore size of 10 µm. This process aimed to remove low-molecular-weight gel agglomerates that are by-products of the reaction. Subsequently, the samples were degassed under vacuum

The obtained prepolymer solutions in different amide solvents were investigated to determine their molecular characteristics. The molecular weights of the polyamic acids were determined using gel permeation chromatography (GPC). Table 1 presents data obtained during GPC measurements.

### 2.5. Film Preparation

PAA solutions were cast onto the hydrophobized surfaces of glass substrates to obtain equally thick PI film samples of approximately 40 μm. The samples were dried at 80 °C for 12 h and cured in stepwise heating regimes to obtain the final PIs. To investigate the effect of the PI film formation process, we subjected the PAA coatings to different heat treatment (imidization) regimes in programmable thermostats.

To investigate the effects of thermal curing on the electrical strength and thermophysical properties of the final PI films, different modes of imidization of polyamic acids were chosen. Two thermal imidization regimes were used for each series of the synthesized polyamic acid structures. For the R-ODA prepolymer (PAA), the first imidization mode was 100, 200, 300 °C for 1 h and the second mode was 100, 200, 300 °C for 1 h, and then 330 °C for 15 min. For the ODPA-ODA prepolymer (PAA), the first imidization mode was 100, 200, 300 °C for 1 h and the second mode was 100, 200, 300 °C for 1 h and then 340 °C for 15 min. For the PMDA-ODA prepolymer (PAA), the first imidization mode was 100, 200, 300 °C for 1 h and then 360 °C for 15 min and the second mode was 100, 200, 300 °C for 1 h and then 380 °C for 15 min.

For short-term electrical strength studies, polyamic acid solutions were further diluted with amide solvents to achieve a concentration of 5% by weight, starting from initial concentrations of 15% and 20%. Additionally, samples with an initial concentration of 5% polyamic acid solution were prepared. All samples were cast onto hydrophobized glass substrates measuring 10 × 10 cm^2^, and subsequently cured using previously described thermal imidization methods.

**Figure 2:**
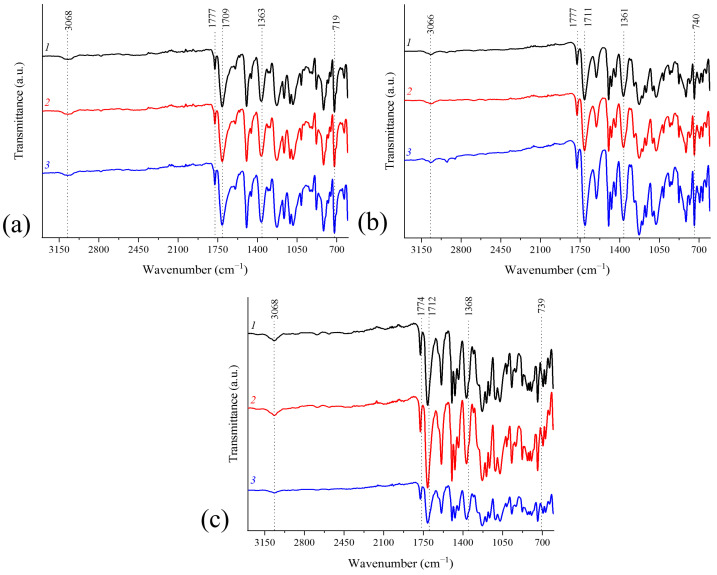
IR spectra of PMDA-ODA (**a**), ODPA-ODA (**b**), and R-ODA (**c**) samples (1-synthesis of PAA in DMF, 2-synthesis of PAA in DMAc, and 3-synthesis of PAA in NMP). R-ODA, IR spectrum: 3068 cm^−1^ C-N aromatic, 1774 and 1712 cm^−1^ C=O imide, 1368 and 739 cm^−1^ C-N imide; ODPA-ODA, IR spectrum: 3066 cm^−1^ C-H aromatic, 1777 and 1711 cm^−1^ C=O imide, 1361 and 740 cm^−1^ C-N imide; PMDA-ODA, IR spectrum: 3068 cm^−1^ C-H aromatic, 1777 and 1709 cm^−1^ C=O imide, 1363 and 719 cm^−1^ C-N imide.

Infrared (IR) spectroscopy was used to analyze the chemical structure of the obtained series of PIs. The IR spectra of laboratory samples of PIs formed from PAA solutions in various amide solvents are presented in Figure 2.

## 3. Results and Discussion

### 3.1. Polyimide Chemical Synthesis

**Figure 3:**
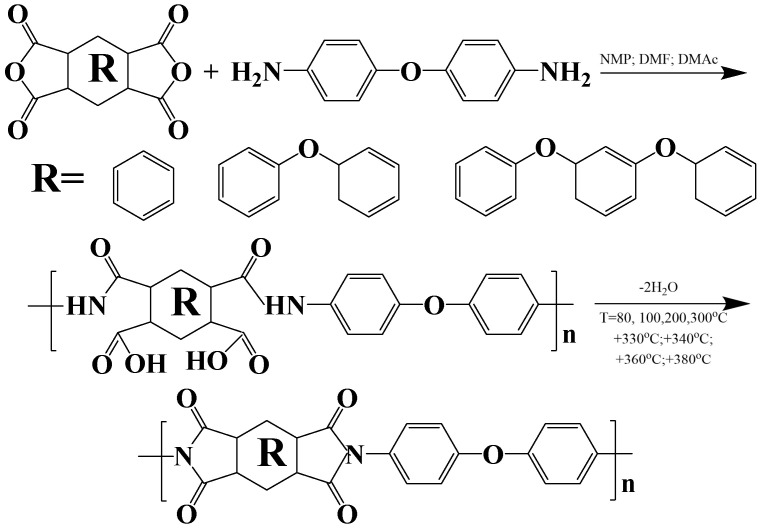
Scheme of the two-stage polyimide chemical synthesis.

R-ODA, ODPA-ODA, and PMDA-ODA polyimides have oxygen atoms in their structure that act as hinges (Figure 1). PIs based on R and ODPA dianhydrides are characterized by the availability and processability with respect to the techniques used in injection molding and additive technologies (3D printing, plasma powder spraying, and a number of others). Homologues of polyimide R-ODA are currently obtained as pre-powders capable of 3D processing and pressure molding. Films of laboratory samples of PIs were prepared according to a known two-stage scheme: first, through the formation of an intermediate product of polycondensation of polyamic acid; second, its thermal cyclization into PI (Figure 3).

The main objective of this work was to determine how the electrical properties change when the conditions for preparing PI samples are altered, such as the choice of amide solvent and the final temperature of the curing of prepolymers (imidization of polyamic acids). In our opinion, these parameters of PI film preparation processes have a significant influence on the electrical properties due to changes in the physical structure of the films.

According to the GPC data presented in Table 1, the molecular weight of polyamic acids reaches its maximum when dimethylacetamide is used as an amide solvent. The analyzed samples exhibited a monomodal molecular weight distribution, with polydispersity indices ranging from 1.5 to 2.5, characteristic of polymers synthesized via polycondensation processes.

All PIs had similar monomer compositions based on IR spectroscopy data (Figure 2). Prepared under different synthesis conditions, they possess a comparable chemical structure and consequently exhibit similar vibrational spectra [45]. The bands of C=O valence vibrations of the amido acid group (amide I, 1650–1660 cm^−1^), N-H strain vibrations (amide II, 1535 cm^−1^), and O-H stretching vibrations (o-carboxyamide group, 1410 cm^−1^) were absent, indicating a high degree of imidization in all samples. This is extremely important for the performance properties of the synthesized polymers. The symmetric (1778 cm^−1^) and asymmetric (1714 cm^−1^) valence vibrations of the C=O bond in the imide cycles are characteristic of PIs. The effects observed in the IR spectra align with the concept of imidization of PIs [46].

### 3.2. Study of the Specific Density of PIs

It was interesting to investigate the density changes of PI films under different thermal imidization regimes. Density measurements were made on PI films using the flotation method by equilibrating the samples in a mixture of carbon tetrachloride and toluene [44]. The results for the PI films imidized at 300, 330, 340, 360, and 380 °C are presented in Table 2.

Based on the data presented in Table 2, it can be stated that the specific density of PIs varies: it increases in the cases of ODPA-ODA and PMDA-ODA series PIs and decreases in the case of R-ODA. Thus, it can be concluded that as the temperature of imidization increases, there is a decrease in free volume and compaction of the imide structure of the film samples from the PMDA-ODA and ODPA-ODA series. This does not occur for samples from the R-ODA series. The investigation of free volume in the considered thermoplastic PIs will be conducted in future studies.

### 3.3. X-Ray Diffraction Study

**Figure 4:**
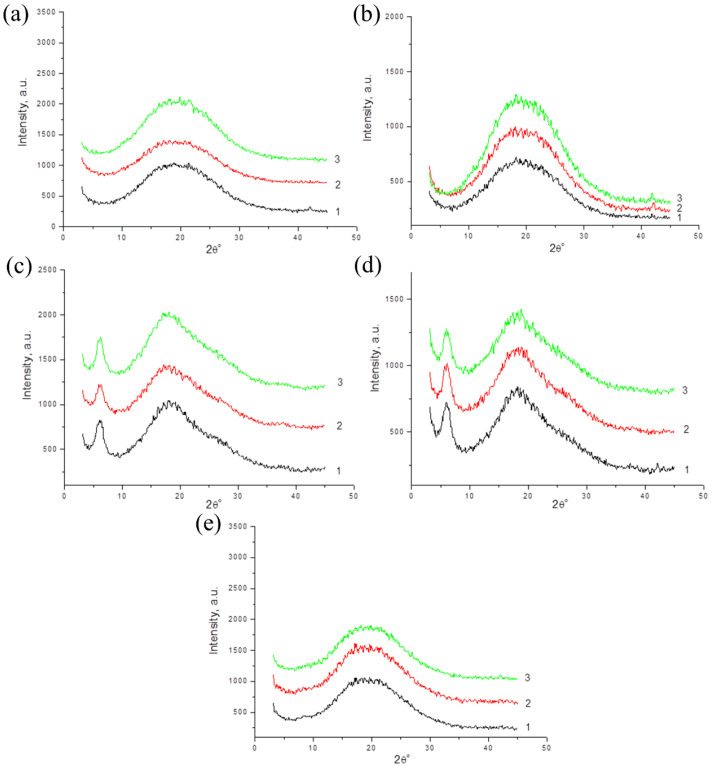
X-ray diffraction patterns obtained for polyimide samples that were subjected to different imidization temperatures: (**a**) maximum of 300 °C and (**b**) 340 °C for ODPA-ODA, with three different synthesis conditions: (1) PAA in NMP, (2) PAA in DMAc, and (3) PAA in DMF; (**c**) 360 °C and (**d**) 380 °C for PMDA-ODA, with the same three synthesis conditions; (**e**) 300 °C for R-ODA, with the same three synthesis conditions.

To reveal the crystal structure of the synthesized PIs, wide-angle X-ray scattering (WAXS) was used for PI samples cured at imidization temperatures up to 300 °C for R-ODA, up to 300 and 340 °C for ODPA-ODA, and 360 and 380 °C for PMDA-ODA. Figure 4 shows the obtained X-ray diffraction patterns of the investigated PIs.

X-ray diffraction analysis showed that after the imidization of polyimides up to 300 °C for ODPA-ODA and up to 300 °C for R-ODA, these PI samples remained in an amorphous state (an amorphous halo was present in the X-ray diffraction images). At the same time, in R-ODA polyimide after imidization at 300 °C, a crystalline structure began to form, although the size of the crystallites formed was too small, judging by the large half-width of the overlapping X-ray maxima. In the imidization regime of PIs up to 340 °C for ODPA-ODA, the appearance of the amorphous halo did not change significantly, whereas for PMDA-ODA, at temperatures up to 380 °C, we can assume some improvement in ordering (a slight increase in the size of crystalline regions). The crystal structure of PMDA-ODA has not been identified so far (no data from the literature are available); therefore, a more detailed study of its crystal structure will be the aim of our future studies.

### 3.4. Thermophysical Properties

Polyimide films cured at 300 °C for a series of R-ODA and ODPA- ODA samples and at 360 °C for a series of PMDA-ODA samples were investigated by thermogravimetric analysis (TGA). The samples cured at 330, 340, and 380 °C showed similar results. The TGA data and calculated values of the thermal resistance indices (τ_5_, τ_10_) are presented in Table 3 and Figures S1–S9 in the Supplementary Materials. The TGA curves were identical regardless of the imidization regime. From the TGA data obtained in an inert atmosphere, it can be seen that the main mass loss region is in the range of 520–640 °C for R-ODA and ODPA-ODA series samples, while for PMDA-ODA series compositions it is slightly higher than 580–680 °C, which is logical considering the properties of this high-temperature-resistant PI known in the literature [3,4,5,6,7,47]. Thermal stability was evaluated using the mass loss indices at 5% (τ_5_) and 10% (τ_10_). It can be noted that the thermal stability indices of R-ODA and ODPA-ODA films tested in an inert atmosphere are very close: τ_5_ in the range of 546–551 °C and τ_10_ in the range of 566–572 °C. For PMDA-ODA samples, an increase in the values of heat resistance indices is observed: τ_5_ in the range of 580–586 °C and τ_10_ in the range of 595–600 °C; therefore, one should take into account the type of solvent when investigating the heat resistance of PIs.

**Figure 5:**
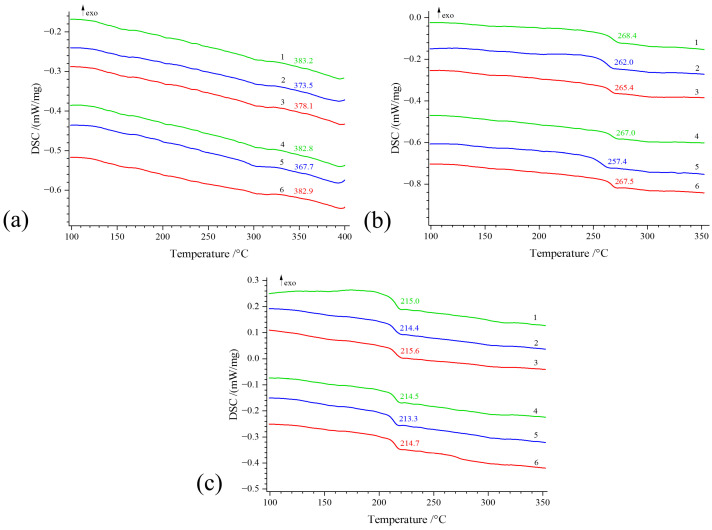
Results from a second series of DSC scans of polyimide films. (**a**) PMDA-ODA, (**b**) ODPA-ODA, and (**c**) R-ODA with varying parameters: different solvents DMAc 1,4, DMF 2,5, and NMP 3,6 and different imidization temperatures (**a**) 360 °C 1,2,3, 380 °C 4,5,6, (**b**) 300 °C 1,2,3, 340 °C 4,5,6, and (**c**) 300 °C 1,2,3, 330 °C 4,5,6.

According to the results of the DSC analysis, the glass transition temperatures of the R-ODA, ODPA-ODA, and PMDA-ODA samples were 214.4–215.6 °C, 262.0–268.4 °C, and 373.5–383.2 °C, respectively. A relationship between the chemical structure and molecular mobility of polyimides was observed. An increase in the number of oxygen atoms acting as hinges in PI macromolecules helped to reduce their glass transition temperature. The DSC curves of the second scan for the glass transition temperatures of the PI samples cured in different imidization regimes are shown in Figure 5.

It is worth noting that determining the glass transition temperature for PMDA-ODA samples using DSC is challenging due to the rigidity of the PI macromolecule. In the figures, the increase in heat capacity does not exceed 0.1 J/(g×K), so further determination of the glass transition temperature for PMDA-ODA should be conducted using dynamic mechanical analysis (DMA)

DSC investigations showed that all films exhibited kinks related to the glass transition temperature with no pronounced exo- and endothermic effects in the high-temperature region, indicating that the samples were amorphous. Notably, a small crystalline phase is observed in the R-ODA sample cured at 330 °C. The TGA revealed that PMDA-ODA films are more thermally stable than R-ODA and ODPA-ODA films.

**Figure 6:**
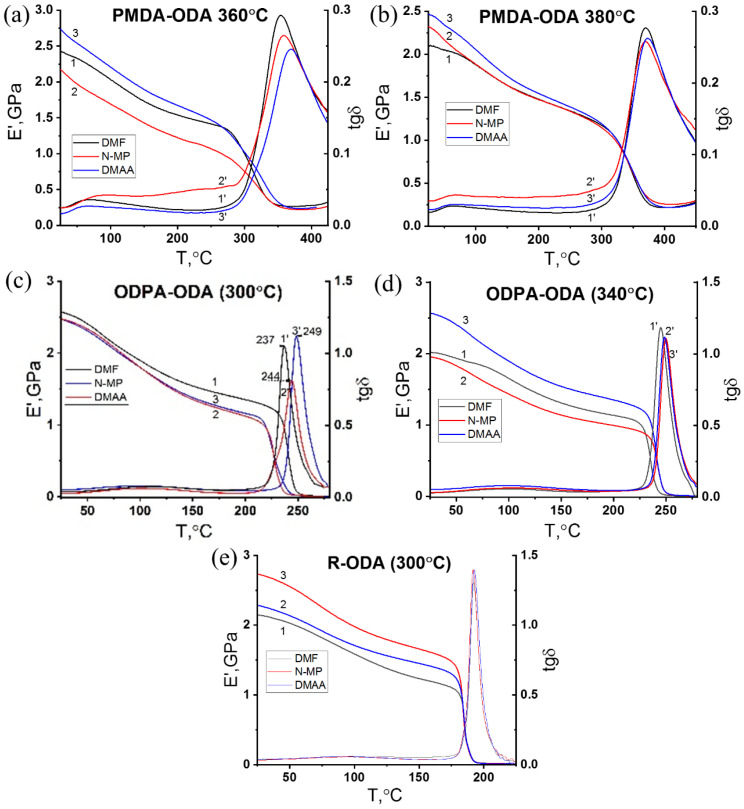
Temperature dependence of E′(T) (1,2,3) and tgδ(T) (1′,2′,3′) measured by DMA for polyimide films derived from various solvents (DMF, NMP, DMAc): (**a**) PMDA-ODA with imidization at 360 °C, (**b**) PMDA-ODA with imidization at 380 °C, (**c**) ODPA-ODA with imidization at 300 °C, (**d**) ODPA-ODA with imidization at 340 °C, and (**e**) R-ODA with imidization at 300 °C.

DMA was used to investigate the relaxation processes of PI films. The experimental DMA data are presented in Figure 6 and Table 4.

The glass transition temperature of the PIs was determined from the high-temperature maximum tgδ(T) and the decrease in the elastic modulus E′(T). The glass transition temperatures as a function of solvent and imidization regimes are presented in Table 4.

The tgδ(T) profiles clearly showed two relaxation maxima (Figure 6) for all studied PIs in the examined temperature range. At 100 °C, a broad relaxation process is observed, attributed to the unfreezing of small chain regions in the diamine and dianhydride components of the PI macromolecule. The high-temperature peaks in tgδ(T) are related to the unfreezing of the segmental mobility of PI macromolecules, which defines the glass transition temperature. As the stiffness of PI macromolecular segments increases, this maximum shifts to higher temperatures [48].

Notably, the choice of amide solvent used in the synthesis of polyamic acids affected the glass transition temperature, particularly for ODPA-ODA and PMDA-ODA polyimides. The highest *T_g_* values were observed when DMAc was used as a solvent for their polyamic acids (see Table 4). In contrast, under our selected laboratory synthesis conditions, DMF-based polyimides exhibited lower *T_g_* values due to poorer molecular mass gain (Table 1), as shown in Table 4.

As shown in Figure 6a,b, the glass transition temperatures of PMDA-ODA determined from the maxima of tgδ(T) correlated well with the molecular weight of PMDA-ODA. The higher the molecular weight, the higher the glass transition temperature. The ODPA-ODA polyimide obtained in DMF had the lowest molecular weight (19,500 g/mol) and consequently the lowest glass transition temperature (237.0 °C, 245.1 °C), in line with ODPA-ODA in DMAc and NMP solvents. The molecular weights of R-ODA in different solvents were sufficiently close; therefore, the position of the glass transition temperature was not affected. The presence and number of atoms that could be considered hinged groups within a polymer chain substantially affect molecular movement and intermolecular interactions. This has a direct impact on the glass transition temperature. It can be seen that for all investigated PI films, additional imidization heating resulted in an increase in the glass transition temperature, as determined by the DMA method through the maximum in temperature dependence of the loss tangent. The analysis of DMA data and the density measurements (Table 2) of PIs suggest that higher imidization temperatures lead to increased density for ODPA-ODA and PMDA-ODA. However, for the R-ODA series, this effect appears to reverse, which is probably associated with the processes of thermodestruction of PI during imidization at elevated temperatures. According to the DSC data of the PIs of the R-ODA series at higher imidization temperatures (330 °C), there is partial destruction of the PI macromolecule.

For the entire range of polyimides studied, a substantial decrease in the elastic modulus was observed in the area where segmental mobility within the PI polymer chains begins to increase. The glass transition temperatures in Table 4 were identified from the drop in elastic modulus regions. The highest molecular weights of PMDA-ODA and ODPA-ODA synthesized in DMAc correspond to high elastic moduli, approximately 2.5 GPa, as illustrated in Figure 6b,d. There was no correlation found between the molecular weight and elastic modulus in the case of R-ODA. The correlation between the elasticity and molecular weight models depends strongly on the chemical structure and flexibility of the polymer chain and on the ability of the polymer to form oriented regions due to the contribution of π–π interactions between benzene rings.

### 3.5. Mechanical Properties

The obtained film coatings under different curing conditions were tested by stress–strain analysis, and the results are summarized in Table 5.

The PMDA-ODA films were stretched uniformly, with no local deformations. In contrast, a clearly defined maximum indicating the yield point of R-ODA and ODPA-ODA is observed on the deformation curves (Figures S10–S12 in the Supplementary Materials). This behavior is typical of flexible-chain polyimides. A so-called neck forms during stretching, whose propagation throughout a sample leads the deformation beyond the yield point.

Analysis of the obtained data showed that the imidization carried out at higher temperatures contributed to an increase in the strength and elongation at break of almost all synthesized polyimides. The influence of the solvent on the mechanical properties was also significant. Depending on the desired characteristics of the material, this should be taken into account.

### 3.6. Short-Term Electrical Strength Study

Therefore, determination of the breakdown strength (E_b_) is necessary for the development of new and modified polymer materials. The prolonged use of insulating PI materials is usually followed by breakdown. However, a breakdown is also possible in the case of overvoltage during product operation.

The value of E_b_ determined in a homogeneous electric field or with increasing radius of curvature of the corona electrode increases. The following results should be considered when comparing different conditions for the synthesized PIs.

The main objective of this work was to study the short-term electrical strength of PIs based on their history of production: synthesis, monomer composition, and imidization temperature conditions. It is known that dilute solutions of polymers are used for coating electronics and elements in electrical engineering. Therefore, the peculiarities of the technology for forming electrically insulating coatings in electronics and electrical engineering were considered. Solutions of PAA ranging from 5% to 20% in amide solvents were prepared, cured, and thermally imidized under the conditions described above. The maximum cyclization temperature of PAA for the R-ODA series varied from 300 to 330 °C, for the ODPA-ODA series from 300 to 340 °C, and for the PMDA-ODA ones from 360 to 380 °C.

No traces of sliding discharge were observed on the surface within the contour of the breakdown channel. It can be assumed that an electrical breakdown occurred. The dependence of the electrical strength at a constant voltage and the resistivity over a wide temperature range was previously studied [49]. It follows from the obtained data that thermal breakdown occurs at temperatures above 200 °C. In the current study, electrical strength measurements were carried out at room temperature, and the results indicated that electrical breakdown occurred.

To confirm this, the electrical strength of the PI films was tested at constant voltage in a wide temperature range [49]. Thermal breakdown was found to occur at temperatures above 200 °C. Table 6 lists the values of E_b_ in the Weibull coordinates.

Table 6 shows that the breakdown field E_b_ values for the initial PIs were in the range of 131 to 258 kV/mm. The highest E_b_ values were observed for the PMDA-ODA and ODPA-ODA films. The electrical strength of PIs is influenced by the chemical structure of the molecules, including the length of the free-membered chain, solution concentration, and the imidization temperature. The chemical structure of a sample exerts a complex influence on its short-term electrical strength. Regardless of the type of solvent used in the synthesis of PIs, the presence of oxygen atoms as hinges in their chemical structure leads to a decrease in electrical strength. Conversely, findings reported in [50] demonstrate that incorporating SO_2_ polar hinged atom groups leads to an increase in E_b_, even when the glass transition temperatures are comparable. Consequently, it remains challenging to definitively ascertain the factors affecting E_b_. Further research is needed to compare the electrical strength of polyimides with varying chemical structures across a wider range of changes in hinged atom groups.

**Figure 7:**
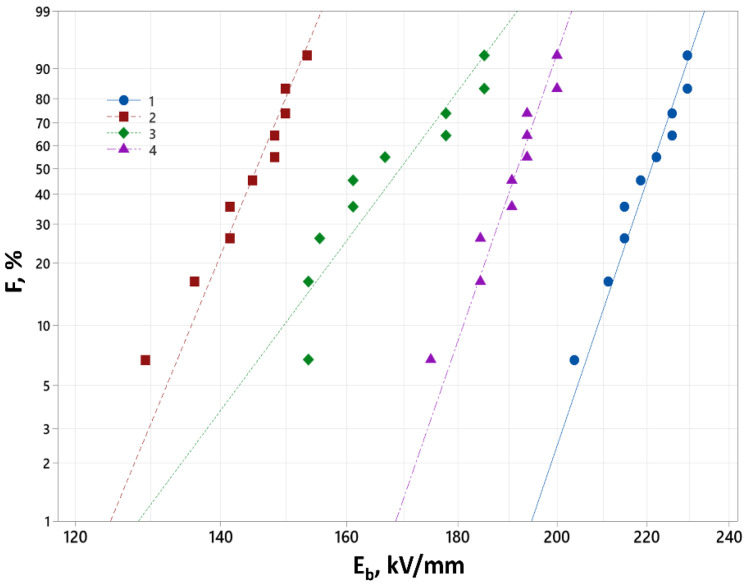
The distribution function F(N) of breakdown field E_b_ plotted in Weibull coordinates for R-ODA films synthesized with NMP solvent. Preparation conditions for the films included: (1) a 5% solution, imidization at 300 °C; (2) a 20% solution, imidization at 330 °C; (3) a 20% solution, imidization at 300 °C; and (4) a 5% solution, imidization at 330 °C.

The dependence of the breakdown probability F(N) on E_b_ for R-ODA is shown in Figure 7. The obtained values of E_b_ for the investigated samples were derived by the Weibull distribution.

When the concentration of the PAA solution decreased from 20% to 5%, the breakdown field E_b_ increased for all the PIs studied. The influence of the type of amide solvent on the short-term electrical strength of each of the synthesized PIs had a distinct character. With increasing imidization temperature, the short-term electrical strength of the ODPA-ODA and PMDA-ODA films obtained from a dilute solution (5%) tended to increase, while that of the PMDA-ODA and R-ODA films (initially at 15%) tended to decrease. The conditions of PI production have a complex effect on the short-term electrical strength of PIs, which requires further detailed study.

The E_b_ values of ODPA-ODA were close to those of the PMDA-ODA films, indicating that this work resulted in a thermoplastic PI whose short-term electrical strength was not inferior to that of PMDA-ODA.

## 4. Conclusions

In this study, polyimides with different chemical structures based on ODA diamine and PMDA, ODPA, and R dianhydrides were investigated. The synthesized series of PMDA-ODA polyimides was selected as a benchmark for comparison with thermoplastic polyimides. The use of dianhydrides with a large number of aromatic rings, as well as oxygen atoms in the structure, during the synthesis of polyimides allows for the creation of promising thermoplastic materials for modern electrical and electronic equipment. In this study, the influence of the chemical structure of polyimides and the solvent used in the synthesis of PAA on their mechanical and thermal characteristics was examined. The obtained results showed that the solvents studied in this work (DMAc, DMF, and NMP) influence the mechanical properties of polymers, leading to an increase in the glass transition temperature and thermal stability of the polymer films depending on the imidization mode. It was found that the imidization modes effected changes in the specific density of the laboratory films, while there was no significant impact on their morphological features, which indicates different structural ordering of polyimides in various solvents and at different annealing modes (final imidization temperature and duration).

The influence of annealing conditions and amide solvents used in the synthesis of PAA on the free volume changes in the films has been shown. The short-term electrical strength of the synthesized series of polyimides was investigated. This value is related to the complex properties of the chemical structure of polyamic acids and the modes of formation of polyimide film material. A promising series of films based on the thermoplastic polyimide matrix ODPA-ODA was obtained, whose short-term electrical strength is comparable to that of the laboratory polyimide PMDA-ODA.

Thus, this study provides a basis for acquiring fusible PIs and their composites, which can be used as perspective materials for 3D printing, plasma spraying, extrusion molding, and films without nanopores. These polymers can be used as elements in capacitors and electrical and electronic equipment and can replace PMDA-ODA, which cannot be processed using modern plastic processing methods. Utilizing the experimental data generated in this research, computational models of the thermal, mechanical, and electrical characteristics of thermoplastic polyimides can be developed and simulated.

## Figures and Tables

**Table 1:** Molecular masses of PAA obtained by GPC.

Sample PAA	M_n_, g/mol	M_w_, g/mol	Ð = M_w_/M_n_
PMDA-ODA(DMAc)	42,600	69,000	1.62
PMDA-ODA (DMF)	21,300	52,400	2.46
PMDA-ODA (NMP)	40,500	67,900	1.67
ODPA-ODA (DMAc)	17,000	34,000	1.99
ODPA-ODA (DMF)	10,000	19,500	1.94
ODPA-ODA (NMP)	16,200	29,900	1.84
R-ODA (DMAc)	33,600	52,300	1.56
R-ODA (DMF)	27,800	45,800	1.65
R-ODA (NMP)	31,500	50,100	1.59

**Table 2:** Density values of polyimides subjected to imidization at different final temperatures.

Samples of solvent used in the synthesis of PAA	Density ρ, g/cm^3^	
	Imidization temperature 360 °C	Imidization temperature 380 °C
PMDA-ODA in DMAc	1.398	1.415
PMDA-ODA in DMF	1.399	1.410
PMDA-ODA in NMP	1.408	1.411
Samples of solvent used in the synthesis of PAA	Density ρ, g/cm^3^	
	Imidization temperature 300 °C	Imidization temperature 340 °C
ODPA-ODA in DMAc	1.356	1.369
ODPA-ODA in DMF	1.354	1.371
ODPA-ODA in NMP	1.355	1.369
Samples of solvent used in the synthesis of PAA	Density ρ, g/cm^3^	
	Imidization temperature 300 °C	Imidization temperature 330 °C
R-ODA in DMAc	1.370	1.354
R-ODA in DMF	1.370	1.354
R-ODA in NMP	1.371	1.353

**Table 3:** Results from differential scanning calorimetry (DSC) and thermogravimetric analysis (TGA) of PIs synthesized in different solvents at various maximum imidization temperatures.

Sample and Solvent PAA	Imidization Up to °C	T_m_, °C	ΔH, J/g	*T_g_*, o 2 Scan	Residual Weight, %	τ_5_, °C	τ_10_, °C
PMDA-ODA in DMAc	360	-	-	383.2	60.9	586	600
	380	-	-	382.8			
PMDA-ODA in DMF	360	-	-	373.5	61.4	580	595
	380	-	-	376.7			
PMDA-ODA in NMP	360	-	-	378.1	59.5	583	598
	380	-	-	382.9			
ODPA-ODA in DMAc	300	-	-	268.4	59.0	551	572
	340	-	-	267.0			
ODPA-ODA in DMF	300	-	-	262.0	59.3	550	572
	340	-	-	257.4			
ODPA-ODA in NMP	300	-	-	265.4	59.4	550	572
	340	-	-	267.5			
R-ODA in DMAc	300	-	-	215.0	58.5	546	566
	330	316.1	<0.3	214.5			
R-ODA in DMF	300	-	-	214.4	59.1	550	568
	330	316.9	5.8	213.3			
R-ODA in NMP	300	-	-	215.6	58.7	550	568
	330	316.4	1.5	214.7			

**Table 4:** Dynamic mechanical analysis (DMA) data of the obtained PIs under different thermal imidization conditions.

Solvent	*T_g_* (tgδ_max_), °C				
	PMDA-ODA (360 °C)	PMDA-ODA (380 °C)	ODPA-ODA (300 °C)	ODPA-ODA (340 °C)	R-ODA (300 °C)
DMF	354.0	370.0	237.0	245.1	192.1
NMP	359.0	370.0	249.0	250.0	191.7
DMAc	369.0	372.8	244.0	248.6	192.6
*T_g_* (E′), °C					
Solvent	PMDA-ODA (360 °C)	PMDA-ODA (380 °C)	ODPA-ODA (300 °C)	ODPA-ODA (340 °C)	R-ODA (300 °C)
DMF	319.0	349.0	240.0	235.4	184.8
NMP	323.0	348.7	225.0	241.7	183.8
DMAc	328.0	351.1	226.0	240.6	184.8

**Table 5:** Strain–strength characteristics of PIs annealed under different conditions during thermal cyclization.

Sample and Solvent PAA	Imidization Up to °C	Young’s Modulus E, GPa	Strength at Break σ, MPa	Strain at Break ε, %
PMDA-ODA in NMP	360 °C	2.26 ± 0.01	97 ± 6	17 ± 2
	380 °C	2.40 ± 0.20	151 ± 14	80 ± 6
PMDA-ODA in DMF	360 °C	1.97 ± 0.08	107 ± 7	40 ± 5
	380 °C	2.47 ± 0.03	128 ± 9	63 ± 7
PMDA-ODA in DMAc	360 °C	1.76 ± 0.05	90 ± 5	27 ± 2
	380 °C	2.14 ± 0.30	168 ± 10	93 ± 13
ODPA-ODA in NMP	300 °C	2.52 ± 0.16	109 ± 6	17 ± 3
	340 °C	2.61 ± 0.03	137 ± 11	55 ± 5
ODPA-ODA in DMF	300 °C	3.17 ± 0.11	126 ± 11	14 ± 3
	340 °C	2.90 ± 0.02	165 ± 9	50 ± 3
ODPA-ODA in DMAc	300 °C	3.02 ± 0.11	149 ± 10	103 ± 7
	340 °C	2.61 ± 0.02	125 ± 7	32 ± 5
R-ODA in NMP	300 °C	2.74 ± 0.15	109 ±11	23 ± 4
	330 °C	2.32 ± 0.15	89 ± 6	33 ± 7
R-ODA in DMF	300 °C	2.29 ± 0.15	69 ± 10	16 ± 4
	330 °C	2.78 ± 0.26	131 ± 13	11 ± 4
R-ODA in DMAc	300 °C	2.33 ± 0.12	97 ± 4	18 ± 4
	330 °C	2.46 ± 0.15	107 ± 2	84 ± 9

**Table 6:** Breakdown field E_b_ of PI films formed from different concentrations of polyamic acid solutions and imidized under different thermal regimes.

Sample	Solution concentration, %	Solvent	Electrical strength, kV/mm	
			Imidization up to 360 °C	Imidization up to 360 °C
PMDA-ODA	5	DMAc	222 ± 8	239 ± 8
	15	DMAc	207 ± 18	184 ± 12
	5	DMF	197 ± 14	253 ± 25
	15	DMF	252 ± 23	195 ± 15
	5	NMP	238 ± 14	200 ± 32
	15	NMP	258 ± 29	199 ± 19
Sample	Solution concentration, %	Solvent	Electrical strength, kV/mm	
			Imidization up to 300 °C	Imidization up to 340 °C
ODPA- ODA	5	DMAc	250 ± 24	237 ± 14
	20	DMAc	196 ± 10	191 ± 13
	5	DMF	192 ± 15	225 ± 16
	20	DMF	182 ± 9	131 ± 11
	5	NMP	193 ± 8	244 ± 31
	20	NMP	175 ± 8	191 ± 15
Sample	Solution concentration, %	Solvent	Electrical strength, kV/mm	
			Imidization up to 300 °C	Imidization up to 300 °C
R-ODA	5	DMAc	216 ± 17	210 ± 19
	20	DMAc	173 ± 11	143 ± 5
	5	DMF	178 ± 10	184 ± 6
	20	DMF	147 ± 6	195 ± 18
	5	NMP	223 ± 8	194 ± 7
	20	NMP	173 ± 13	147 ± 7

## Data Availability

The data presented in this study are available upon request from the corresponding authors.

## Supplementary Materials

The following supporting information can be downloaded at https://www.mdpi.com/article/10.3390/polym17101385/s1. Figures S1–S9. Temperature dependence of TGA and DTG for polyimides PMDA-ODA, ODPA-ODA, and R-ODA film prepared on DMAc, DMF, and NMP. Figures S10–S12. Stress–strain dependence of PMDA-ODA, ODPA-ODA, and R-ODA polyimide films prepared in different solvates (DMAc, DMF, and NMP) and cured at different temperatures.
