# Supplementary material for: The Effect of Synthesis Conditions and Chemical Structure of Thermoplastic Polyimides on Their Thermomechanical Properties and Short-Term Electrical Strength
Source: Polymers (Basel). 2025 May 18;17(10):1385. doi: 10.3390/polym17101385 (PMC12115213; doi:10.3390/polym17101385)
Supplement: Supplementary file 1 [file polymers-17-01385-s001.zip › polymers-3625954-supplementary.pdf]

Supplementary Materials

for

The Effect of Synthesis Conditions and Chemical Structure of Thermoplastic Polyimides on Their Thermomechanical Properties and Short-Term Electrical Strength

Victor M. Nazarychev[1,*], Andrey A. Pavlov[2], Almaz M. Kamalov[1], Margarita E. Borisova[2], Andrei L. Didenko[1,*], Elena M. Ivan'kova[1], Vadim E. Kraft[1], Gleb V. Vaganov[1], Alexandra L. Nikolaeva[1], Anna S. Ivanova[1], Victor K. Lavrentiev[1], Elena N. Popova[1], Ivan V. Abalov[1], Aleksey N. Blokhin[1], Alexander N. Bugrov[1,3] and Vladislav V. Kudryavtsev[1]

[1] Branch of Petersburg Nuclear Physics Institute named by B.P. Konstantinov of National Research Centre «Kurchatov Institute»—Institute of Macromolecular Compounds, Bolshoi, pr. 31 (V.O.), 199004 St. Petersburg, Russia;

[2] Peter the Great St Petersburg Polytechnic University, the Politekhnicheskaya St. 29, St. Petersburg 195251, Russia;

[3] Department of Physical Chemistry, Saint Petersburg Electrotechnical University (ETU "LETI"), ul. Professora Popova 5, 197376 St. Petersburg, Russia;

* Correspondence: nazarychev@imc.macro.ru (V.M.N.); vanilin72@yandex.ru (A.L.D.); Tel. +7-(812)-3230216

1. Thermal gravimetric analysis (TGA) and first derivative of the TGA (DTG).

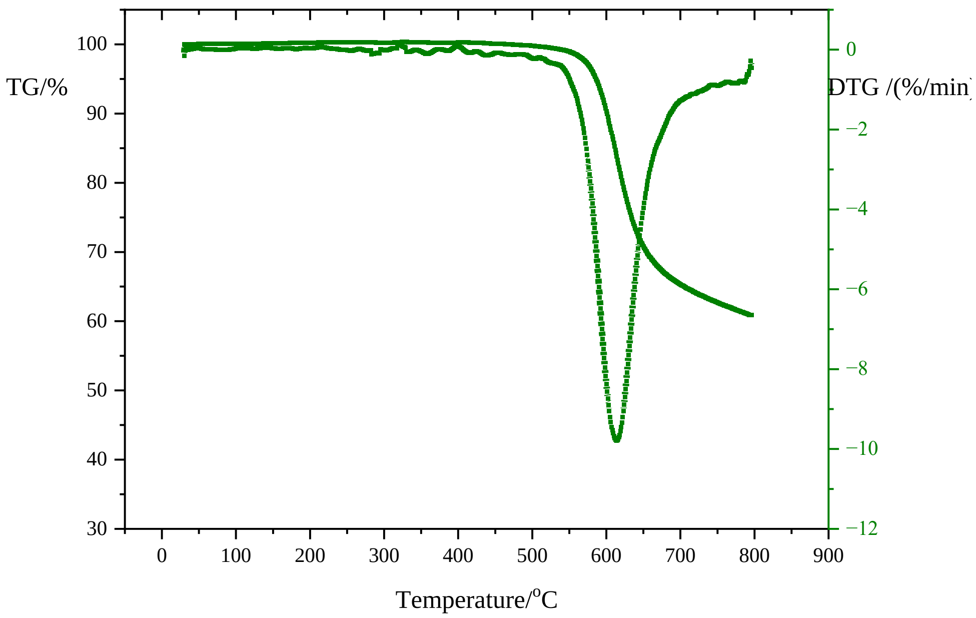

Figure S1. The temperature dependence of TGA and DTG for PMDA-ODA film prepared on DMAc.

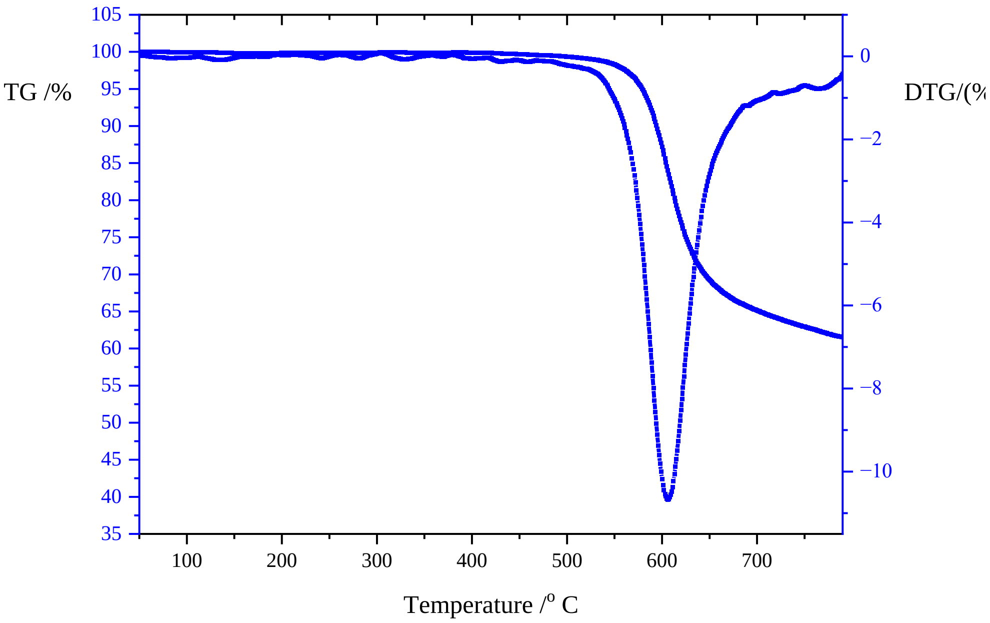

Figure S2. The temperature dependence of TGA and DTG for PMDA-ODA film prepared on DMF.

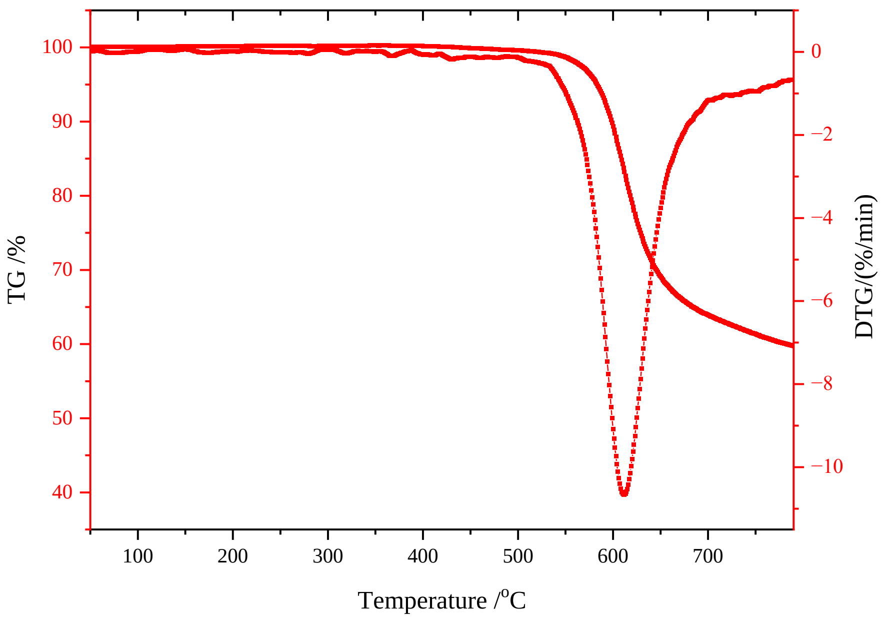

Figure S3. The temperature dependence of TGA and DTG for PMDA-ODA film prepared on NMP.

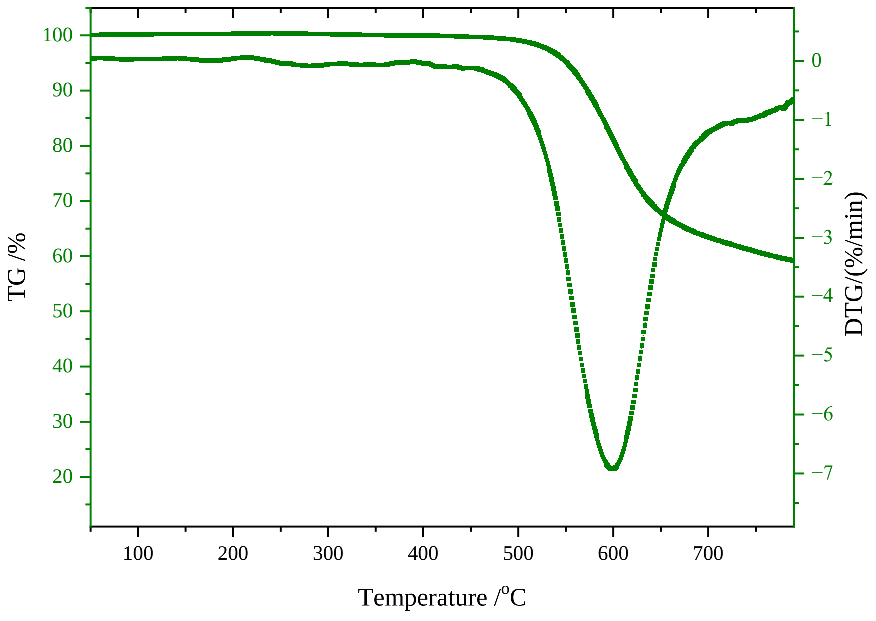

Figure S4. The temperature dependence of TGA and DTG for ODPA-ODA film prepared on DMAc.

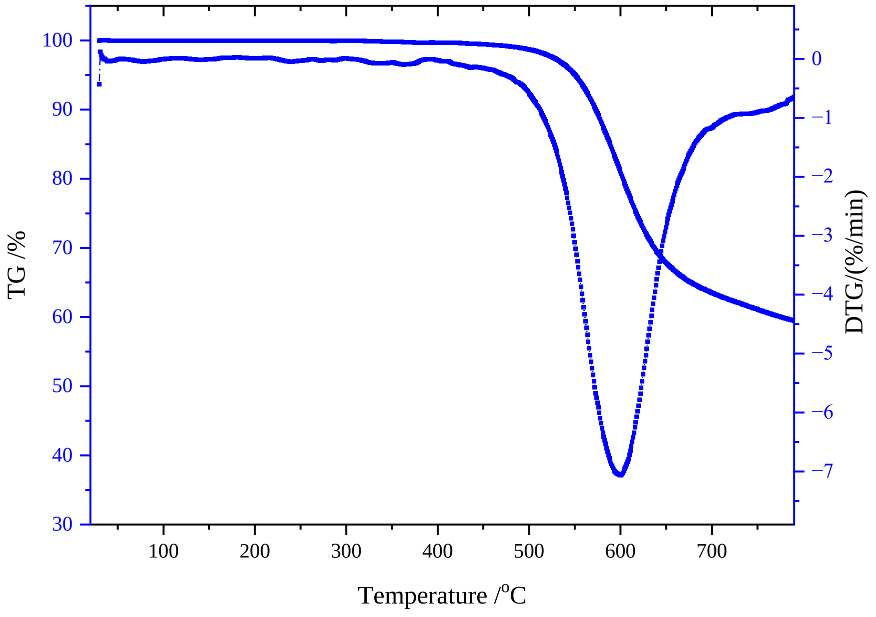

Figure S5. The temperature dependence of TGA and DTG for ODPA-ODA film prepared on DMF.

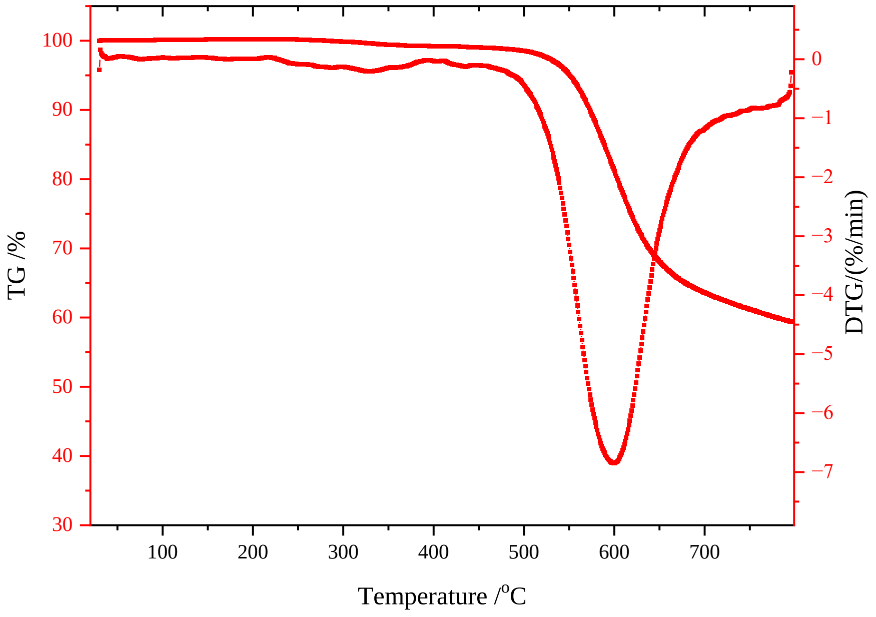

Figure S6. The temperature dependence of TGA and DTG for ODPA-ODA film prepared on NMP.

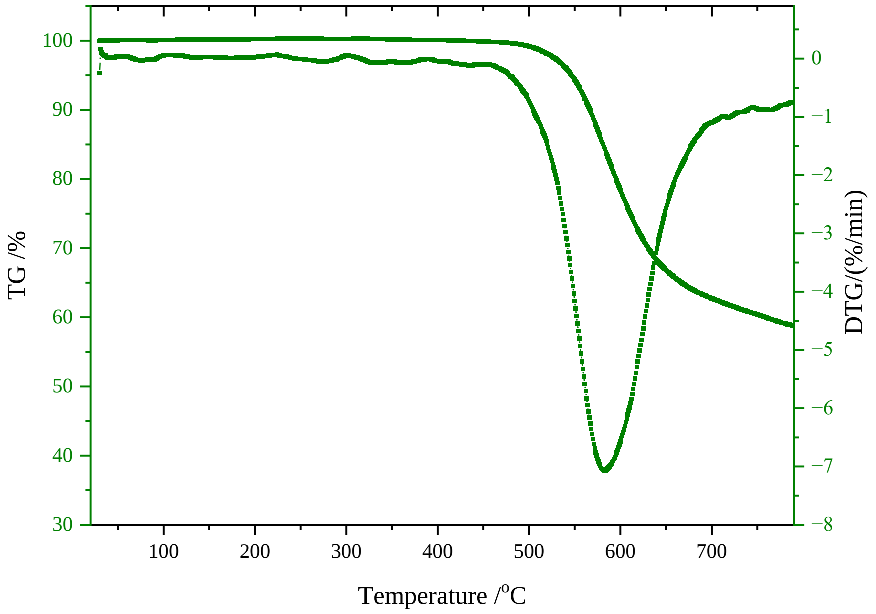

Figure S7. The temperature dependence of TGA and DTG for R-ODA film prepared on DMAc.

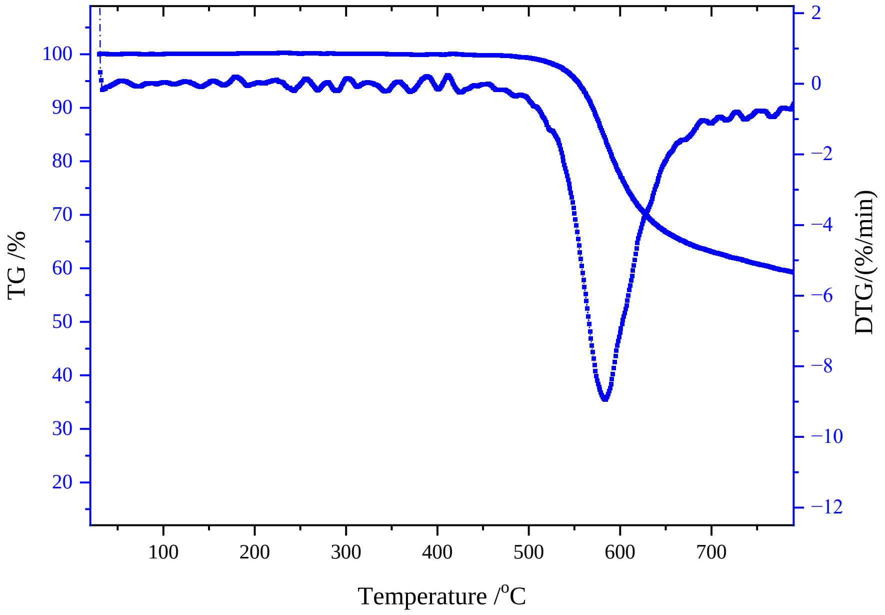

Figure S8. The temperature dependence of TGA and DTG for R-ODA film prepared on DMF.

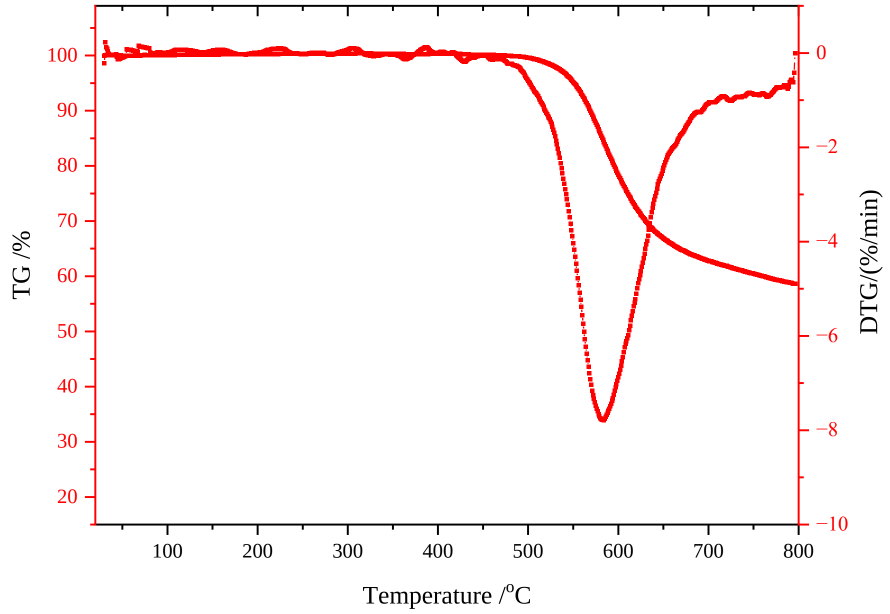

Figure S9. The temperature dependence of TGA and DTG for R-ODA film prepared on NMP.

2. Stress-strain dependencies of polyimide films.

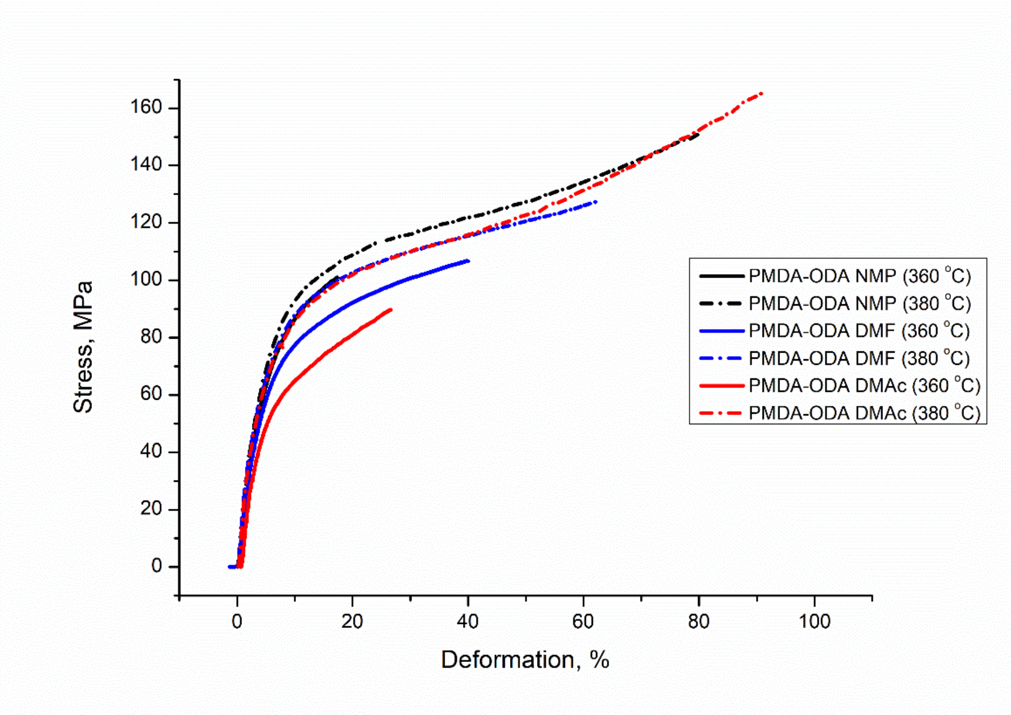

Figure S10. Stress-strain dependence of PMDA-ODA polyimide film prepared in different solvates (DMAc, DMF, and NMP) and cured at different temperatures (360 °C and 380 °C).

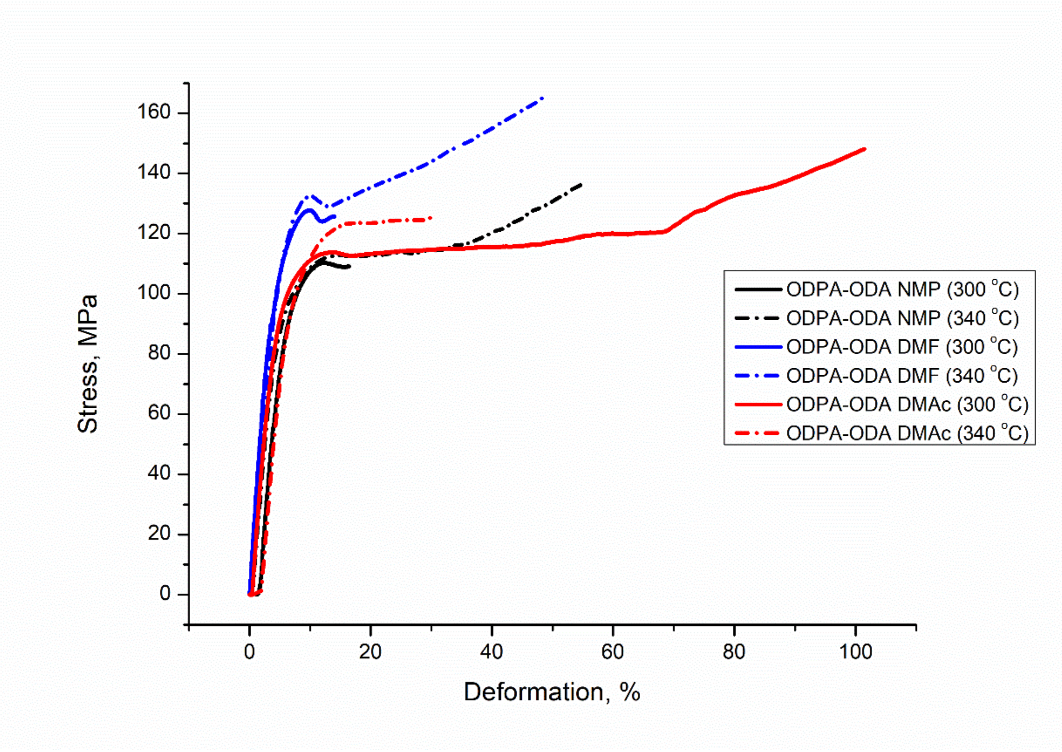

Figure S11. Stress-strain dependence of ODPA-ODA polyimide film prepared in different solvates (DMAc, DMF, and NMP) and cured at different temperatures (300 °C and 340 °C).

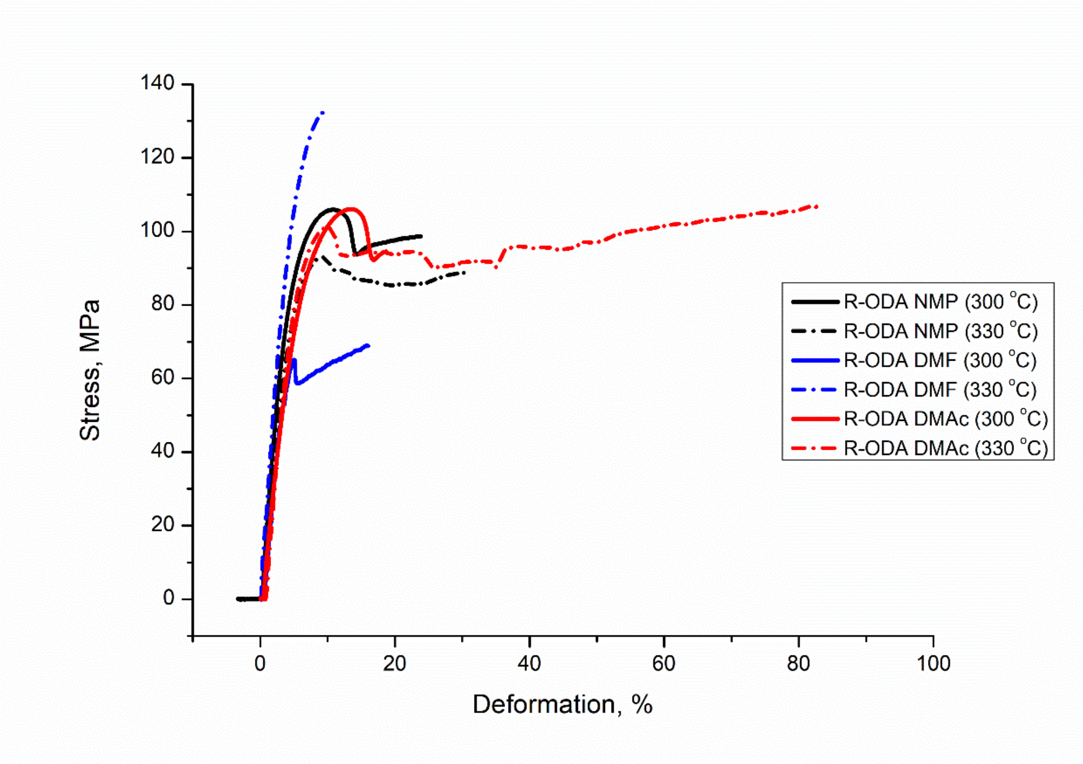

Figure S12. Stress-strain dependence of R-ODA polyimide film prepared in different solvates (DMAc, DMF, and NMP) and cured at different temperatures (300 °C and 330 °C).
